# Achieving universal health coverage in South Africa through a district health system approach: conflicting ideologies of health care provision

**DOI:** 10.1186/s12913-016-1797-4

**Published:** 2016-10-07

**Authors:** Adam Fusheini, John Eyles

**Affiliations:** 1 Centre for Health Policy/MRC Health Policy Research Group, School of Public Health, University of the Witwatersrand, Private Bag X3, Wits, 2050 Johannesburg, South Africa; 2 School of Geography and Earth Sciences, McMaster University, Hamilton, Ontario Canada

**Keywords:** Universal Health Coverage, District Health System, Inequity, Ideology, South Africa

## Abstract

**Background:**

Universal Health Coverage (UHC) has emerged as a major goal for health care delivery in the post-2015 development agenda. It is viewed as a solution to health care needs in low and middle countries with growing enthusiasm at both national and global levels. Throughout the world, however, the paths of countries to UHC have differed. South Africa is currently reforming its health system with UHC through developing a national health insurance (NHI) program. This will be practically achieved through a decentralized approach, the district health system, the main vehicle for delivering services since democracy.

**Methods:**

We utilize a review of relevant documents, conducted between September 2014 and December 2015 of district health systems (DHS) and UHC and their ideological underpinnings, to explore the opportunities and challenges, of the district health system in achieving UHC in South Africa.

**Results:**

Review of data from the NHI pilot districts suggests that as South Africa embarks on reforms toward UHC, there is a need for a minimal universal coverage and emphasis on district particularity and positive discrimination so as to bridge health inequities. The disparities across districts in relation to health profiles/demographics, health delivery performance, management of health institutions or district management capacity, income levels/socio-economic status and social determinants of health, compliance with quality standards and above all the burden of disease can only be minimised through positive discrimination by paying more attention to underserved and disadavantaged communities.

**Conclusions:**

We conclude that in South Africa the DHS is pivotal to health reform and UHC may be best achieved through minimal universal coverage with positive discrimination to ensure disparities across districts in relation to disease burden, human resources, financing and investment, administration and management capacity, service readiness and availability and the health access inequalities are consciously implicated. Yet ideological and practical issues make its achievement problematic.

## Background

There is a growing enthusiasm for universal health coverage (UHC) at global and national levels. It has emerged as a silver bullet solution to health care needs in low and middle income countries [1]. Currently, UHC is at the center of current efforts to strengthen health systems and improve the level and distribution of health and health services [2].

Beyond generating attention globally and nationally, UHC has also been set as a possible umbrella goal for health in the post-2015 development agenda [3, 4]. But the WHO and the World Bank see financial protection, service sustainability and equity as defining features [5]. In their case study of 11 countries, Maeda et al. noted the different stages in developing and implementing UHC with a goal being the coverage obtained after many years in mature economies such as France and Japan [5]. Several countries, including Ghana and Vietnam, are at various implementation stages and while much response is positive, there remain issues of population coverage and out-of-pocket expenses in Ghana [6] and capitation payments and hospital operational autonomy in Vietnam [7]. South Africa is currently reforming its health system with UHC through developing a national health insurance (NHI) program and is in a similar position to Ghana and Vietnam, cases which show how other health reforms and local context can affect UHC implementation [6]. The NHI represents a substantial policy shift that will necessitate a massive reorganisation of the current health care system, both public and private. Importantly it derives its mandate from the National Development Plan (NDP) of the country, blueprint for the shape of South African society in 2030.

As a health financing system, the NHI is designed to pool funds to provide access to quality, affordable personal health services for all South Africans based on their health needs, irrespective of their socioeconomic status. Thus, the NHI is intended to ensure that the use of health services does not result in financial hardships for individuals and their families [8]. According to the Government’s White Paper on the NHI, in South Africa, the implementation of the NHI is consistent with the Constitutional commitment for the state to take reasonable legislative and other measures, within its available resources, to achieve the progressive realization of the right to all for access to health care services including reproductive health care. Progressively, realizing this right will contribute to a healthy population that benefits the entire nation. Therefore, the NHI is a policy shift that will contribute towards poverty reduction and address the inequalities inherited from the past. Under the NHI, population coverage will ensure all South Africans have access to comprehensive quality health care services. This implies that people will be able to access health care services closest to where they live. The health care services will be accessed at the appropriate level of care and will be delivered through certified and accredited public and private providers using the NHI Card [8].

Despite the global and national momentum, however, the paths of countries to UHC have differed with varying health systems [9] and reform efforts. But it has been argued that UHC is likely to remain an empty promise unless it is focused on the provision of quality essential services to everyone by strengthening local health systems [10]. But as Ghana and Vietnam show, much depends on local context, including other health reforms. South Africa’s path to UHC is complicated by not only its history but also the size of the private health sector and its present political complexion, still dominated by liberation ideology and the importance of solidarity and inclusion. Local involvement is seen as central for this and thus the district health system is a key component in reform efforts at UHC. District Health Systems (DHSs) emphasize the importance of organizing and coordinating health service delivery at the local level as the strategy embodies a decentralized, area-based, people-centered approach to health care [11].

In Africa, the DHSs’ strategy has become the backbone of nearly every national health system. Countries are covered by health facilities-organized in a tiered system [10]. Even when new financial mechanisms are brought into play as in Burundi [12], the district is seen as a necessary component allowing the local implementation of new service delivery models. In this paper, we explore the opportunities and challenges of the district health system in achieving UHC in South Africa. However, it has been shown that South Africa is some way away from UHC [13]. To carry out this commentary, we first examine UHC and its ideological premises and then link UHC to the provision of services at the local level. The reality of local service availability is then discussed, showing variable provision (inequities) between districts. So, the question is why the district? Is its policy attraction ideologically different from that of UHC and are they reconcilable? We explore National Health Insurance (NHI) pilot districts as an attempt at practical reconciliation, with the district as a potential element for positive discrimination. In this section, we examine what constitutes UHC and the proposed features of South Africa’s NHI.

### Universal health coverage

There is confusion as to what UHC actually is [1]. Broadly defined, it means all people receiving the health services they need, including health initiatives designed to promote better health (such as anti-tobacco policies), prevent illness (such as vaccinations), and to provide treatment, rehabilitation, and palliative care (such as end-of-life care) of sufficient quality to be effective while at the same time ensuring that the use of these services does not expose the user to financial hardship [2, 14, 15]. This resonates with the government’s White Paper on the NHI, which will cover a comprehensive set of health services that will provide a continuum of care from community outreach, health promotion and prevention to other levels of care [8]. The comprehensive package of health services delivered will cover (but not limited to) the following: preventive, community outreach and promotion services; reproductive health services; maternal health services; pediatric and child health services; HIV and AIDS; and tuberculosis services; health counselling and testing services; chronic disease management services; optometry services; speech and hearing services; mental health services including substance abuse; oral health services; emergency medical services; prescription medicines; rehabilitation care; palliative services; diagnostic radiology and pathology services [8].

Meanwhile, it is instructive to mention that UHC comprises two main components: quality, i.e. essential health service coverage, and financial coverage – both extended to the whole population. Three dimensions underlie countries’ efforts in progressing towards UHC as identified by the World Health Organization. These are population coverage, service coverage and cost/financial coverage [14]. This resonates with the philosophical underpinnings of international development agencies (IDAs), that see UHC as a health financing system based on pooling of funds to provide health coverage for a country’s entire population, often in the form of a ‘basic package’ of services made available through health insurance and often provided by a growing private sector [1].

In other words, UHC is defined in terms of rights to health care, financial protection, and utilization of health care services on an equitable basis [16]. Universal coverage thus implies *equity of access* and *financial risk protection*. Its defining feature is a prescription of a clear split between health financing and health provision, allowing for the entry of private insurance companies, private health providers and private health management organizations [1]. Does this prescription imply a specific ideological commitment? The right to health care is enshrined in the South African constitution. Yet for over twenty years since the government of national unity, it was not until December 2015 that the African National Congress (ANC) government issued a white paper on the implementation of a NHI, which is seen as a step towards achieving UHC in South Africa. The strengthening of the DHS is, therefore, seen as an integral part of the preparation towards achieving UHC by making health services more accessible and available, especially, to the rural poor; and for fulfilling the constitutional mandate or requirement. According to the Government’s White Paper on the implementation of the NHI, the first phase extends from 2012/2013 to 2016/2017 financial years. This phase of implementation has focused on Primary Health Care (PHC) facilities in some DHS, seen as pilots.

### Proposed features of the South African NHI

In terms of population coverage, NHI will extend coverage to all South Africans irrespective of their socio-economic status. Coverage will also extend to legally permanent residents. In extending effective population coverage (i.e. ensuring that those in need can in reality access quality services), priority will be given to the population that is in greatest need and must include those experiencing the greatest difficulty in obtaining care. The identification of the population with the greatest need will be based on criteria consistent with the principles of NHI. Vulnerable groups will be prioritized [8].

NHI will have a single-payer mandatory prepayment mechanism where resources are pooled in a single fund to cater for the health needs of the entire population via a strategic purchaser. By this, the NHI will ensure that individuals and households do not suffer financial hardship and/or are not deterred from accessing and utilizing needed health services. It involves eliminating various forms of direct payments such as user charges, co-payments and direct out-of-pocket payments to accredited health service providers. In effect, the NHI aims at providing a comprehensive package of health services to all South Africans according to need and not ability to pay [8]. The White Paper on the implementation of the NHI identifies potential sources for funding the scheme as including: direct and indirect taxation, payroll tax and collection of premiums or membership contributions from employees or informal sector [8]. The document is, however, silent on how much premium is to be paid. In terms of cost, a preliminary policy paper issued by the government estimated that NHI will cost R255 billion (~US$30 billion) per year by 2025, if implemented as planned over a 15-year period [17].

## Methods

So given this background, how is UHC evolving within a district-based system? An extensive literature review conducted to gather relevant descriptive and quantitative data on UHC and the DHS formed the main methodological approach for investigating this. The main focus was on UHC and its ideology, the link between UHC and service availability, service availability and districts and the focus on districts using the case of the NHI pilots in South Africa. As part of the literature review process, an assessment of relevant publications including government policies, Department of Health (DoH) reports, District Health Barometer (DHB), South African Health Review, working papers and academic articles concerning the topic were also made use of.

Thus materials were collected from different and multiple sources including documents, policy briefs, reports, bulletins and academic papers as a way of triangulation so as to give credibility and dependability [18] to the study. This also ensured verification and cross checking of the information gathered. A systematic and manual literature search was conducted across several electronic databases in the social and health sciences, as well as on Google scholar to locate peer-reviewed studies published on UHC and the DHS. Major databases searched using Keyword Identifiers were ProQuest Central, Applied Social Science Index and Abstracts (ASSIA), MEDLINE, International Bibliography of the Social Sciences (IBSS), and Web of Science. A broad systematic search of UHC in ProQuest Central, for instance, returned 23,689 original peer reviewed studies. The search terms were further defined and combined to improve the search. Thus, the search was narrowed to Africa, which returned 6392 peer reviewed studies and then South Africa (5086 results). Of these, fifteen were used in relation to policy and policy change in South Africa. “The District Health System” revealed 70,923 results in ProQuest databases. A search of the district health system in South Africa returned 13,637 results of which nine studies focus specifically on the DHS in low and middle income countries. The searches took place between September 2014 and December 2015. We also reviewed and examined documents including text books, annual reports of the DoH, consultancy reports, health sector independent reviews, technical reports, websites and other grey literature. Twenty of this type of material was used in the study.

## Results and discussion

Reviewing national and international documents as well as the academic literature on DHS and UHC and their ideologies, we explore the prospects for UHC in South Africa.

### UHC’s ideology of neoliberalism

Ideologically, the goal of UHC, whether tax- (likely NHI route) or insurance-based is deeply ingrained in a broadly-defined politics, ethics and international law [19]. While UHC has been of great appeal to societies with strong social democratic and labor movements [20], “countries cannot simply spend their way to universal health coverage. To sustain progress, efficiency and accountability must be ensured; and the main health financing instrument for promoting efficiency in the use of funds is purchasing and more specifically, strategic purchasing” [21]. Purchasing must be related to defined population health needs and access to services, however, they are provided. Proposed UHC models of implementation are not neutral and can allow for the use of private sector resources [22], thus lending itself to standard neoliberal policies (value for money, consumer choice, market-based decision-making), steering policy-makers away from universal health options based on public systems with the state generally confined to the role of system manager [1]. Health thus becomes a marketable commodity as neoliberal ethos becomes dominant in most of these countries health systems reforms. The consequence of this is that equity and efficiency are compromised due to ideological pressures that prevent the adoption of an entirely public system of care provision (ibid). In Mexico, for example, patients must pay out-of-pocket for using private doctors. In 2015/16, it is estimated that while South Africa spends 8.5 % of Gross Domestic Product (GDP) on health, 4.1 % of the GDP is spent on 84 % of the population, the majority utilizing the public health sector whilst 4.4 % of its GDP is spent on only 16 % of the population [8]. This shows the inequality in access to quality health services between the upper 16 % of the population and the remaining 84 %. Thus, financing through medical schemes and Out-of-Pocket payments (OOPs); accounts for a significant proportion of health care financing; which is beyond the reach of the majority of South Africans. The expenditure on medical schemes in South Africa is more than in any Organization for Economic Cooperation and Development (OECD) country and represents more than six times the 2013 OECD average of 6.3 %. This type of a financing system disadvantages the poor and those working in the informal sector and leaves many citizens at a high risk of financial ruin due to catastrophic health expenditure. Besides, it is maintained that health care benefits are not distributed in line with the need for health care services. The benefit incidence of health care in South Africa is very ‘pro-rich’, with the richest 20 % of the population receiving 36 % of total benefits (despite having a ‘health need share’ of less than 10 %) while the poorest 20 % receive only 12.5 % of the benefits (despite having a ‘health need share’ of more than 25 %). Preliminary estimates also indicate that the contribution by government to medical schemes (open and restricted) in 2015 is well-in-excess of R20 billion annually and these funds are mostly spent within the private health sector [8]. Within the private health sector, members of medical schemes are subjected to high OOPs. According to the Council for Medical Schemes annual report, OOPs increased by 11.9 % to R20.7 billion between 2013 and 2014. This translates to approximately R6,000 per beneficiary (8.8 million covered beneficiaries) paid out as OOP for accessed services. These figures, according to the Council for Medical Schemes are an understatement of OOPs as beneficiaries do not claim for all OOPs when they realize that their medical scheme will not reimburse them for these OOPs [8]. These call for fairer financing mechanism and more increase government role if UHC is to be achieved in South Africa.

Indeed evidence shows that all countries that have achieved UHC have done so with an increase government role in the financing, regulation, and sometimes direct provision of health care services [9, 23–27]. In South Africa, UHC is to be achieved through NHI, the pooling of resources and risk through taxation. Yet since 2011 there has been a fierce ideological debate between public health support and the large private sector, in which over half of all health expenditures occur for about 16-18 % of the population. Will a public construction of UHC through NHI enhance service availability?

### Can UHC provide service availability?

If the main goal of universal health coverage is to ensure good quality care for all then universal access to services is a precondition to achieving universal coverage [4, 28]. But the inequities between districts with respect to population health status is well-known. For example, it has been pointed out that rates in the most impoverished districts in the country are double that of the least impoverished districts in terms of HIV prevalence [29]. For instance, in an antenatal client survey (ANC) conducted in 2012, KwaZulu-Natal (KZN) had the highest prevalence (37.4 %), followed closely by Mpumalanga (35.6 %) and Free State (32.0 %). Western and Northern Capes had the lowest prevalence rates of 16.9 and 17.8 % respectively. The picture at the districts level is similar to the provincial figures with HIV prevalence ranging from 1.5 % in Namakwa (Northern Cape) to 40.7 % in uMgungundlovu (KZN), the latter being one of the NHI pilot districts. However, five NHI pilot districts had prevalence rates below the national average of 29.5 %: Eden (Western Cape), Vhembe (Limpopo), Pixley ka Seme (Northern Cape), Tshwane (Gauteng), and Dr Kenneth Kaunda (North West). Among the NHI pilot districts, uMgungundlovu (KZN), Gert Sibande (Mpumalanga) and Thabo Mofutsanyana (Free State) reported the highest HIV prevalence of 40.7, 40.5 and 33.5 % respectively [30]. As yet there is little research on these district differences with more attention being paid to racial and provincial ones [13].

But with these differences emerging more clearly, there needs, therefore, to be differential improvements in services. Can this be through UHC and what is the relationship between UHC and service availability? O’Neill et al. identify two types of service availability-general and service-specific. General Service availability is concerned with the physical presence of items required for the delivery of services and encompasses health infrastructure, core health personnel and aspects of service utilization. On the other hand, service-specific availability is concerned with whether a specific type of health intervention is offered. Intervention may be defined by target population, for example, pregnant women, infants, children, the poor, HIV positive patients, and by specific programme [28]. Although South Africa is not one of their cases, O’Neill et al. identify variations between districts in, for example, Zambia with greater availability and readiness for HIV-related services in 2010 compared with 2008 but declines in trained staff. Overall, however, more districts have higher levels of availability and readiness. But this research is important as it shows that availability and readiness varies over time and requires close attention. Thus variations at the district level show that UHC may have practical challenges as well as ideological ones.

### What is the link between service availability and districts?

District health systems comprising primary health care and first referral hospitals, are key [31] to the delivery of basic health services in developing countries [32]. The district health system (DHS) is often seen as the means of achieving an equitable, efficient and effective health system based on the principles of the Primary Health Care (PHC) approach [33]. This is because a main strength of the health district model is the combination of strong values - equity, efficacy, efficiency, autonomy and solidarity with conceptual neatness and operational relevance [34]. These values are key principles of NHI systems and the DHS model is, therefore, seen as a key element of UHC in South Africa. It is argued that being more than a form of organization; the DHS depicts a set of activities such as community involvement, integrated and holistic health care delivery, intersectoral collaboration and a strong “bottom-up” approach to planning, policy development and management [34]. This has been a central plank of the South African health system since 1997 [31].

The link between service availability and the district cannot be overemphasized as the DHS model applies to the whole health system and at all levels of healthcare delivery. For example, although about 90 % of South Africans live within seven kilometers of a public sector clinic and two thirds within two kilometers, monetary and time-related costs associated with travel to a local clinic can pose considerable barriers to vulnerable populations [35]. Assessments conducted by the Public Services Commission on service delivery inspection of district hospitals and clinics in four provinces (Limpopo, Free State, North West and the Western Cape) showed a number of challenges in relation to service readiness and or availability in South Africa.

In the Free State, staff shortages, infrastructure, and budget constraints were identified as impacting negatively on effective and efficient delivery at the inspected facilities as well as the service readiness of those facilities [36]. In one clinic (Bophelong) the need for additional professional nurses was apparent due to the high patient turn-out [36]. The problem is compounded by nurses going on leave and attendance of meetings or training. This often results in hospitals being forced to utilize staff in other functions in which they may not be trained as is the case in Tokollo hospital [36]. In some districts there is a high labor turnover, especially young professionals. They often feel there is nothing attractive to encourage their long term stay in areas such as Heilbron, (FS). According to the report, lack of amenities such as schools, recreational facilities and accommodation for staff as well as the intermittent power failures have negatively impacted on the functioning of equipment and care of drugs and medical consumables that need to be stored in refrigerators. Furthermore, budgetary constraints, which is not peculiar to one province has impacted on the rollout of certain services such as the RX Solution information system to all districts. At Tokello hospital, for instance, the X-Ray machine needed to be replaced as it has reached its lifespan yet lack of funds implies that the hospital cannot provide quality service.

In the other provinces (Limpopo, North West and the Western Cape) similar issues were present. At the Ellisras Hospital in Limpopo, the report indicated the lack of sufficient doctors as there is poor infrastructure and a poor living environment. In the North West, it was reported that at the Brits Hospital, there was no doctors’ quarters. Other issues noted included infrastructure, generally poor emergency medical services in all the districts due to insufficient ambulances, where response times often range between one and four hours. Sometimes ambulances never arrived after repeated calls placing the lives of affected persons at risk [36]. The Western Cape was characterized by insufficient space to stock pharmaceutical supplies in most clinics, lack of dedicated human resource capacity for pre-packing chronic medication distribution, turnaround time for the disposal and replacement of equipment being unreasonably long at times [36]. And the National Health Care Facilities Baseline Audit National Summary Report showed that “hospitals and PHC facilities throughout the country show a high percentage failure in compliance to the vital measure dealing with the availability of medicines as per the Essential Drug List” [37].

Health human resources vary enormously between provinces. For example, Limpopo and North West have the smallest proportions of health professionals except for registered nurses. In Northern Cape and Mpumalanga, over half of medical practitioner posts are vacant [38, 39]. At the district level, some health provider groups are almost or completely non-existent. Writing about surgery in rural areas, Chu comments: “Tintswalo does not have an anesthesiologist, and the postoperative recovery room is not equipped to handle postoperative complications such as cardiac arrhythmias or respiratory distress. Intravenous cardiac inotropes and vasopressors are unavailable. There is no intensive care unit. The surgical ward nurses are not trained in the postoperative care of patients after major procedures. During the night, there is often not a single professional nurse staffing the surgical ward, and the nurse/patient ratio is too high for the acuity of care needed, often exceeding 20:1” [40]. In KZN, most obstetric anesthesia is provided by community service doctors, foreigners and sessionals at the district level [41].

Variations in readiness for service delivery are mirrored by management challenges. Overall, there is variable expenditure on district management. In Eastern Cape, for example, Cacadu district expenditure on management was 10.8 %, while Nelson Mandela Bay spent 5.0 %. In KZN, the percentage of district health services expenditure on district management was lowest in Zululand in KwaZulu-Natal (0.8 %) and highest in Northern Cape in ZF Mgcawu (14.2 %). Eight out of the 10 districts with the lowest percentage expenditure on management were from KwaZulu-Natal, which may point to a lean effective system or lack of capacity.

Yet despite these differences between districts [42], the idea of the district remains strong. It was reinforced in later National Health Acts and expressed well by Van Rensburg, et al. as the district meets the health care needs of all including those in underserved and understaffed areas, in a way that people want to receive their care; it provides a simple, integrated and logical service and thus to overcome the inefficiencies in service delivery caused by undue fragmentation of the system; it ensures that local decisions are made locally, in terms of local needs, and by the very people who have to implement and be affected by the decisions; it involves those people who use the health services in planning and designing their own services by means of fully representative community health bodies; and it shifts the focus from administering health services towards improving health and the quality of care at the local level [43]. It remains fundamental in the re-engineering of primary health with ward-based teams and district clinical specialist teams [33].

### Why districts; a different ideology?

As seen in the preceding section, the focus on districts is underpinned by the fact that in order to achieve equity, the organization of healthcare should be according to geographic sub-divisions of a country, managed through decentralized management structures: a normative statement. Ideologically, the district is related to the need for decentralization and community involvement; and the integration of health programmes. It thus emphasizes the specific characteristics of an area, its particularity. According to the World Bank, decentralization can reduce administrative bottlenecks in decision making and increase the efficiency of government and its responsiveness to local needs. It can also enhance the accountability of public institutions, improve service delivery, and allow greater political representation and participation of diverse groups in decision making [44]. Yet there may be forces working for decentralization and centralization in the same health system as occurs in Brazil [45]. Decentralization is often seen as a response to the drawbacks of large, centralized public institutions, such as poor efficiency, slow innovation, and lack of responsiveness to patients’ preferences. Successful decentralization, however, requires a supportive environment, namely sufficient local administrative and managerial capacity, ideological certainty in the implementation of tasks, and readiness to accept several interpretations of one problem. Experience in a number of countries, particularly in post-communist Europe, shows that when these preconditions are not met, decentralization has negative consequences such as service fragmentation, increased inequity, political manipulation by powerful interests, and a weakening of public-sector regulatory functions [46]. Recentralization may occur [47]. Yet decentralization may increase community involvement and choice [48]. But initially in LMICs, decentralization concerned power sharing, devolved authority and local provision and accountability. There was thus an ideology of solidarity and communitarianism [49] but some suggest a move toward a more choice-based, consumerist perspective [50]. Yet an ideology of solidarity must be maintained in South Africa not only because of its historical and political context but because of the extreme district inequities.

### UHC moving forward in South Africa: pilot districts

A conscious change in policy direction in favor of pursuing UHC in South Africa can be seen in the development and piloting of the NHI in eleven districts since 2012. The aim is to roll out nationwide in a fifteen year period. This can be situated within the context of global trends on desirable health system reforms [51]. The pilots followed the launch of the Green Paper on NHI in August 2011and constituted the first steps towards implementation of a UHC program.

The pilot districts selected are situated in the nine provinces and specifically in areas with high levels of underserved communities. According to Nkosi, selection of the districts was based on health profiles/demographics, health delivery performance, management of health institutions or district management capacity, income levels/socio-economic status and social determinants of health, compliance with quality standards and above all the burden of disease [52]. So can some districts with the lowest levels of service availability be improved with UHC through PHC re-engineering with these targeted NHI funds?

Furthermore, will improving access to quality health services, particularly, in the rural and previously disadvantaged areas require mechanisms for introducing a district health system of funding for health services; and what are the costs of introducing a fully-developed district health authority and implications for scaling up? [52–54]. But what if the pace of roll out slows? How will non-pilot sites respond to their perceived lack of benefit? Does selection (and an emphasis on particularity) limit the significance of national solidarity and communitarianism in providing health care services? These are questions and concerns that need further exploration.

Approximately 12 months after their inception, the Strengthening South Africa’s Response to HIV and Health (SARRAH) conducted an independent review of the current status of the eleven NHI pilot districts and some progress has been made. NHI teams are in post (in nine districts), but only four districts have nationally appointed full-time NHI Project Managers with the other districts making efforts to fill up the various posts. Overall, a significant proportion (about 60 %) of the NHI conditional grant funds has been disbursed by the districts in the last quarter of the year. This has primarily been on procuring equipment and refurbishing health facilities (prior to December 2012, equipping and refurbishing facilities fell outside the remit of the conditional grant; NDoH revised these conditions). There has been progress with District Health Management Teams (DHMTs). Claims of NHI activities being coordinated on a regular basis are also reported. Clinic committees have been established, and most are now functioning. The report further notes progress in hospital reforms with re-designation of district hospitals. Thus full-time CEOs are in post in 60 % of all hospitals. Quality improvement mechanisms have been introduced in 10 of the 11 districts; Facility Improvement Teams (FIT) are said to be functioning, quality assurance plans are being monitored, and Office of Standards Compliance (OSC) assessments are taking place in all the districts. Private GPs were to be contracted in the 2013/14 financial year [53, 54]. Yet incentives for private doctors to work in the public sector are quite limited [55] and although state employment offers a stable income, wages can be six times higher in the private sector, depending partly on hospital department and how much specialist training individuals had undertaken [56]. By December 2015, a total of 555,139 patients were registered in 118 facilities in the selected pilot districts. This system would be functional in all PHC Facilities (698) in the NHI Pilot Districts by 31 March 2016 [8]. As part of the integrated school health program under the NHI, the Department of Health has deployed 70 school mobiles in all pilot districts to provide general, oral health services, eye health services as well as audiology services. The number of learners seen through school mobiles in schools for Grades 1; 4; 8 and 10 were 380,929 in 2013 and 497,933 in 2014 with the following ratios: 19.3 % of Grade 1 learners (during April 2013 to March 2014) and 23.2 % of Grade 1 learners (during April 2014 to March 2015) of Grade 1 learners [8].

Again, contracting of general practitioners to provide PHC services at clinics located within the pilot districts was implemented in the 2013/14 financial year. It has been estimated that over 302 general practitioners have been contracted since. Available data indicates that 152 contracted general practitioners are providing services in 260 PHC facilities in eight pilot districts. Preliminary data indicates that for the 2014/15 financial year, approximately 34,330 patients received services delivered through these general practitioners contributing to the reduction in waiting times and improving access to needed services for the catchment populations served [8].

There remain key challenges in the NHI pilot phase. Overall, there are reports of uneven progress across the districts. The report notes for, instance, that achievements in the Eden (Western Cape), Pixley ka Seme (Northern Cape), Tshwane (Gauteng) and uMgungundlovu Districts (KwaZulu-Natal) were far higher than in the OR Tambo (Eastern Cape) and Vhembe Districts (Limpopo Province). For example, only 2 % of primary healthcare facilities in the OR Tambo and 15 % in the Vhembe Districts were able to provide the full package of primary healthcare services. In contrast, it is claimed that more than 80 % of the facilities in the Eden, 76 % uMgungundlovu, and 65 % in the Pixley ka Seme Districts had sufficient capacity [53]. This situation illustrates the larger human resource capacity disparities across the districts.

There has also been a lack of adequate infrastructural improvement in the pilot districts. Progress has been slow due to poor coordination between the National Department of Health (NDoH) and the Department of Public Works. Preparations for the new NHI services and innovations necessarily depend on improvements to the standard of the available facilities and the coordination of functions between the DoH and the Department of Public Works [53].

Inadequate budget spending guidance from the NDoH was also noted. By the end of the 2012/13 financial year, it was reported that only 77 % of the budget allocated to the pilot districts through conditional grants had been spent. And 90 % of the available funds were used only in the fourth quarter of the financial year [53]. This, as the report admitted, was because the DoH failed to provide clear guidance about which NHI initiatives the funds could be used for. The surge in spending occurred only after the DoH revised its grant criteria. But the transformation will be costly with Treasury estimating 6 billion Rand are required for roll-out each year [57].

There is also the lack of progress in establishing the structures for community participation in primary healthcare service delivery- clinic committees and hospital boards. While all the districts were reported to have hospital boards and clinic committees, there is little detail about how these bodies function and the extent to which they facilitated meaningful community participation. Furthermore, variations in the quality of the structures meant to facilitate public participation were evident. In the Pixley ka Seme District in the Northern Cape, for example, four out of thirty-six clinic committees are described as “functioning optimally” in the report. In contrast, in the OR Tambo District, all the hospitals have boards “but functionality is an issue”. The Gert Sibande District has no protocol for the establishment of clinic committees, and has used the provincial protocol for Hospital Boards instead [53]. In sum, Eagar concludes that NHI piloting needs to prioritise reforms and interventions that promote greater equity, efficiency, effectiveness and participation (i.e. the bases for UHC) [53], which the NHI piloting scheme has largely failed to do so far.

### Does UHC reduce inequities? – Evidence from the pilot districts

The question of whether UHC reduces health inequity remains a question at the present. However, to the extent that UHC removes financial barriers to health care access through the pooling of funds to provide a basic package via health insurance to those who otherwise could not have paid at the point of service, UHC enhances the population access to appropriate promotive, preventive, curative and rehabilitative health care without any catastrophic financial cost. In this regard and in light of state DoH policy, UHC through NHI implies a move towards equity of access and financial risk protection, especially, by which the most excluded and vulnerable populations rise to the same standards of health enjoyed by the more privileged in society. The introduction of community health workers, improvements to monitoring the health of children and decongesting clinics by new modes of chronic illness medication distribution are significant positives.

But as the WHO and Scheil-Adlung et al. note, ‘coverage’ denotes access that is realised, i.e. going beyond legislative entitlement to effective coverage. It also includes the quality of health services received (preventive, promotive, curative, rehabilitative and palliative), social equity, and the financial risk protection that has been obtained [58–60]. But the ability of UHC to reduce health inequlities goes beyond financing, although this is a central and transformative component [61]. Other factors such as availability and location of facilities and their readiness to render services; human resources and their equitable distribution across and within districts; population health status, the health system itself among others are crucial to UHC potential to reducing inequality.

Evidence from the pilot districts demonstrated that out of 301 (49 % of 609) PHC clinics across nine districts assessed in relation to their readiness to deliver comprehensive health care services, one third of them were considered to be ready overall [53] with considerable variation across the districts ranging between 2 % in OR Tambo district and 80 % in Eden district. Even with commitment and conditional funding, existing capacity creates variable improvements between districts and services.

Human resource distribution across the districts is perhaps the outstanding constraint. In the Amajuba district in the KwaZulu Natal Province, the recruitment of the District Clinical Specialist Team (DCST) has proved daunting with the district only being able to recruit the advanced midwife and PHC nurse out of a total membership of seven by the end of 2013. Even with the availability of facilities, UHC might not ensure equity in healthcare access without the required health professionals to deliver services.

### Ideological tension – a minimal universal benefit and the need for positive discrimination

Considered in the context of the disparities across districts in relation to disease burden, human resources, financing and investment, administration and management capacity, service readiness and availability and the inequalities that still exists in the pilot districts, this commentary suggests a need for a minimal universal coverage and for positive discrimination. In line with Brearley et al. contention, financial risk protection, which is at the heart of UHC, has the potential of reducing the vulnerabilities of poor people and strengthening household resilience if the overall system is adequately funded and sufficiently redistributive [61]. Yet there are few pro-poor policies in South Africa [13].

From the results presented above, positive discrimination is already taking place in the pilot districts. Based on principles of solidarity and communitarianism, the worst districts, that were underserved and understaffed, benefit most as South Africa moves towards UHC over a fifteen year period. While discriminating against other districts, bridging the health availability and access gaps between the underserved and better- resourced districts could only lead to improvements in health availability, access and health status outcomes in South Africa. We argue, therefore, that such an approach can be replicated nationwide when NHI is finally implemented. This will ensure resources are freed up in areas that less need them for use in areas that need them- pro-poor, rural and other underserved areas based on health profiles, socio-economic status and service readiness and capability. Such an approach requires dedicated and committed political leadership over the long term, found in the UHC reforms in Brazil and Thailand [5].

Evidence from the data also supports the need for positive discrimination while upholding minimal universal coverage for all South Africans. Yet in a resource-constrained setting, positive discrimination may be resented by those seen as not entitled to these resources, as was found in attracting new staff to underserved areas through a rural allowance. Existing staff felt that they were discriminated against [62]. Resource allocation over the years appears to be in favor of this approach across districts grouped according to socio-economic quintiles. The median percentage of district health services expenditure on district hospitals remained highest in most deprived areas at 50 % compared to around 30 % in less deprived areas. In contrast, median spending on PHC (as a percentage of DHS) in least deprived areas was 66 % compared to 48 % in most deprived areas [30]. So there is a need to spend more on PHC and reduce relatively the expenditure on district hospitals by tackling the social determinants of health (SDoH), which may trickle down to the entire district health system. This process of SDoH impact remains a persuasive but still ill-formed idea in terms of practice changes [63]. But there may not be sufficient resources to provide more funding to health care services. South Africa spends between 8.5 and 9% of GDP on health with a per capita expenditure about one quarter of the OECD average [64]. Much of the increased expenditure in the NHI districts has been in the form of conditional, time-limited grants which may not be affordable for all districts. Conditional grants allow for transfers but must be renewed annually and take account of policy changes [65].

Furthermore, results from the data support the view that positive discrimination for minimal universal coverage at the district level works but sick individuals and communities may not be best served by the geographical approach as there are illnesses in richer areas and healthy people in poorer ones. This scale problem is difficult whatever the UHC implementation solution. For the present there is encouragement and, financially, it is acknowledged that socio-economically deprived districts in South Africa have been receiving more resources than those less deprived ones as a way of ensuring equity and bridging the inequality gaps as “much more is spent on DHS per capita uninsured in deprived areas” [42] than in less deprived areas. Thus, in providing universal health service for all South Africans through NHI, equity is the underlying principle. To ensure equity means discriminating against the richer (and healthier) or those who can afford so as to target the poor and more vulnerable in society. Yet these are the people who must be persuaded to pay health taxes without much personal benefit over a relatively long period of time.

## Conclusions

The paper set out to explore the extent to which the DHS can serve as a catalyst to UHC in South Africa. It noted the differences in population health status and service delivery between districts and noted the possible tensions between the ideologies of UHC and decentralization. Yet there is mixed but largely positive news from the NHI pilots, selected because of poor health conditions and services. Yet there is a concern for the future, financially, ideologically and practically. But in order to ensure no district lags behind in health policy reforms towards UHC, there should be priority setting driven mainly by the objective to achieve equity in access to health and wellbeing outcomes. Service availability and readiness have to be based on the unique conditions of each district, according to the principles of DHS. By emphasizing district particularity, more resources can be committed to those districts that are underserved and in dire need of health facilities and personnel in the country. Nationally, financial sustainability of UHC must be ensured.

Thus in South Africa the DHS is pivotal to health reform and correctly UHC, as defined by WHO, which emphasizes the well-functioning of the entire health system of which the DHS is a part. Whether the financial and ideological challenges will undermine this intent is not known. But as the roll-out of the NHI program to all districts may require a rethinking of the salience of the DHS or a scaling down of UHC developments.

## Abbreviations

ANC: African National Congress
ANC: Antenatal client
ASSIA: Applied Social Science Index and Abstracts
CEO: Chief executive officer
DCST: District Clinical Specialist Team
DHB: District Health Barometer
DHMTs: District health management teams
DHS: District Health System
DoH: Department of Health
FIT: Facility Improvement Team
FS: Free state
GDP: Gross domestic product
GPs: General practitioners
IBSS: International Bibliography of the Social Sciences
IDA: International Development Agency
KZN: Kwa Zulu Natal
LMIC: Low and middle income countries
NDoH: National Department of Health
NDP: National Development Plan
NHI: National Health Insurance
OECD: Organization for Economic Cooperation and Development
OOP: Out-of-pocket payment
OSC: Office of Standards Compliance
PHC: Primary Health Care
SARRAH: Strengthening South Africa’s Response to HIV and Health
SDoH: Social determinants of health
UHC: Universal health coverage
WHO: World Health Organization
